# Novel Observations in 11 Heteroresistant Vancomycin-Intermediate Methicillin-Resistant *Staphylococcus aureus* Strains from South India

**DOI:** 10.1128/genomeA.01425-16

**Published:** 2016-12-22

**Authors:** Yamuna Devi Bakthavatchalam, Balaji Veeraraghavan, John Victor Peter, Janakiraman Rajinikanth, Francis Yesurajan Inbanathan, Naveen Kumar Devanga Ragupathi, Suresh Kumar Rajamani Sekar

**Affiliations:** a Department of Clinical Microbiology, Christian Medical College, Vellore, Tamil Nadu, India; b Division of Critical Care Unit, Christian Medical College, Vellore, Tamil Nadu, India; c Department of General Surgery, Christian Medical College, Vellore, Tamil Nadu, India

## Abstract

We report here the draft genome sequences of 11 heteroresistant vancomycin-intermediate *Staphylococcus aureus* (hVISA) strains from bloodstream infection. All strains harbor mutations in *vraSR*, *graSR*, *walKR*, and/or *tcaRAB* and are often implicated as the frequently mutated candidate genes in hVISA phenotypes.

## GENOME ANNOUNCEMENT

Methicillin-resistant *Staphylococcus aureus* (MRSA) can cause serious community- and nosocomial-acquired infection. The use of vancomycin in the treatment of MRSA infection is challenged by the emergence of heteroresistant vancomycin-intermediate *S. aureus* (hVISA) and vancomycin-intermediate *S. aureus* (VISA). Several studies have reported the increasing frequency of hVISA/VISA-associated treatment failures in invasive infections (1, 2).

Here, we present the draft genome sequences of 11 hVISA strains isolated from bloodstream infections. All the isolates were found to have vancomycin MICs of 1 to 1.5 µg/ml. All these hVISA strains were confirmed with a population analysis profile-area under curve (PAP-AUC) method with the following PAP-AUC ratios: VB988, 1.03; VB9939, 0.92; VB16578, 0.96; VB20017, 1.0; VB44094, 0.97; VB44746, 1.33; VB46389, 1.03; VB35316, 0.96; BA43011, 1.28; *Staphylococcus aureus* strain 2016, 1.02; and VB1490, 1.0.

DNA isolation from pure cultures was performed using QIAamp DNA minikit (Qiagen, Germany). Whole-genome shotgun sequencing was performed using the Ion Torrent PGM system (Life Technologies, Inc.) with 400-bp chemistry. The raw data generated were assembled *de novo* using the assembler SPAdes version 5.0.0.0 embedded in Torrent suite server version 5.0.4. The genome sequence was annotated using PATRIC, the bacterial bioinformatics database and analysis resource (http://www.patricbrc.org) (3), and the NCBI Prokaryotic Genome Annotation Pipeline (PGAP) (http://www.ncbi.nlm.nih.gov/genome/annotation_prok/) (4). Downstream analysis was performed using the Center for Genomic Epidemiology (CGE) server (http://www.cbs.dtu.dk/services) and PATRIC. The resistance gene profile was analyzed using ResFinder 2.1 from the CGE server (https://cge.cbs.dtu.dk//services/ResFinder/) (5). The CRISPR finder (http://crispr.u-psud.fr/Server/) was used to detect and identify clustered regularly interspaced short palindromic repeat (CRISPR) and spacer sequences in the genome. The sequence type was determined for all the isolates in the allele order of *arcc*, *aroe*, *glpf, gmk*, *pta*, *tpi,* and *yqil* by comparing the sequences with *S. aureus* database maintained at the MLST website (http://saureus.mlst.net/).

The annotated genome size of MRSA isolates ranged from ~2.7 to ~2.9 Mb, with coverages of 33× to 88× (Table 1). The number of coding DNA sequences (CDSs) per genome ranged from 2,603 to 3,011. Genome annotation by PATRIC predicted a total of 41 to 60 tRNAs and five to 10 rRNAs in the sequenced isolates (Table 1). All the isolates were found to harbor various toxin and antimicrobial resistance genes. Further, none of the isolates were found to have CRISPR regions.

**TABLE 1:** Genome characteristics of hVISA isolates from bloodstream infection

Isolate ID	Accession no.	Draft genome size (Mbp)	No. of CDSs	No. of contigs	No. of tRNAs	No. of rRNAs	Coverage (×)	Virulence genes^a^	Resistance genes	ST/SCC*mec*/*spa* type^b^
VB9882	MLQI00000000	2.77	2,692	132	60	10	79	*aur, scn, sak, seq, sen,seu, sei,sem, seo, seg, hlb*, *hlgB*, *hlgC*, *hlgA*, *lukS-*PV, *lukF-*PV	*aadD, acc(6′)-aph(2′′), mecA*, *blaZ*, *norA*	2371/I/t6827
VB9939	MLQK00000000	2.83	2,803	437	48	7	33	*aur, scn, seq, sem, seg, seo, sec3, sel, hlb*, *hlgB*, *hlgC*, *hlgA*, *lukS-*PV, *lukF-*PV, *sen,seu, sea/sep*	*ant(6)-la, aph (3′)-III, acc(6′)-aph(2′′), spc, mecA*, *blaZ*, *norA, mph*(C)*, msr*(A), *dfrG*	772/V/t657
VB16578	MLQD00000000	2.79	2,667	143	59	9	61	*aur, scn, seq, sen,seu, sei,sem, seo, seg,sec3, sel, hlb*, *hlgB*, *hlgC*, *hlgA*, *lukS-*PV, *lukF-*PV	*ant(6)-la, aph(3′)-III, acc(6′)-aph(2′′), spc, mecA*, *blaZ*, *norA, mph*(C)*, msr*(A), *dfrG*	772/V
VB20017	MLQE00000000	2.85	2,916	420	41	5	34	*aur, scn, sak, seq, sen,seu, sei,sem, seo, seg, hlb*, *hlgB*, *hlgC*, *hlgA*, *lukS-*PV, *lukF-*PV	*aadD, acc(6′)-aph(2′′), mecA*, *blaZ*, *norA, ermC*	2371/V/ t6827
VB44094	MLQH00000000	2.77	2,683	110	57	10	79	*aur, scn, sak, seq, sen,seu, sei,sem, seo, hlb*, *hlgB*, *hlgC*, *hlgA*, *lukS-*PV, *lukF-*PV	*acc(6′)-aph(2′′), mecA*, *blaZ*, *norA, ermC*	22/IVc/t474
VB44746	MLQF00000000	2.84	2,818	265	50	8	41	*splA*, *splE*, *aur*, *scn*, *sak*, *lukD*, *lukE*, *hlb*, *hlgB*, *hlgC*, *hlgA,eta*	*blaZ*, *norA, ermC*	1290/IVh/t131
VB46389	MLQG00000000	2.80	2,700	235	50	7	42	*aur, scn, sea/sep, seg, seq, sen,seu, sei,sem, seo, seg,sec3, sel, hlb*, *hlgB*, *hlgC*, *hlgA*, *lukS-*PV, *lukF-*PV	*ant(6)-la, aph(3′)-III, acc(6′)-aph(2′′), mecA*, *blaZ, mph*(C)*, msrA*, *norA*, *dfrG*	772/V/t458
VB35316	MLQC00000000	2.77	2,638	88	59	9	85	*splB*, *splA*, *splE, aur, scn, sak*, *lukD*, *lukE, seg, sen,seu, sei,sem, seo, hlb*, *hlgB*, *hlgC*, *hlgA*	*acc(6′)-aph(2′′), mecA*, *blaZ, norA*, *dfrG*	72/III/V/t2473
VB43011	MLQJ00000000	2.77	2,604	67	59	8	76	*splB*, *splA*, *splE, aur, scn, sak, sea/sep, seb, seq, sek,seh*, *lukD*, *lukE, hlb*, *hlgB*, *hlgC*, *hlgA*	*ant(6)-la, aph(3′)-III*, *blaZ, mphC, msrA*, *norA*,	1/V/t127
*Staphylococcus aureus* strain 2016	MLQA00000000	2.72	2,603	85	60	9	88	*splA*, *splE*, *aur*, *scn*, *sak*, *lukD*, *lukE*, *hlb*, *hlgB*, *hlgC*, *hlgA*	*blaZ*, *norA*	580/II/t4615
VB1490	MLQB00000000	2.97	3,011	143	58	8	64	*splB, splA, splE, aur, scn, sak, seq, sek, lukD, lukE, hlb, hlgB, hlgC, hlgA*	*ant(6)-la, aph(3′)-III, acc(6′)-aph(2′′), spc, mecA, blaZ, norA, ermA, tet*(M)	239/III/V/t037

a PV, Panton-Valentine.

b ST, sequence type; SCC*mec*, staphylococcal cassette chromosome *mec* element.

Infection with hVISA/VISA has been associated with high vancomycin MIC and poor clinical outcome. The most frequently mutated two-component system (TCS) determinants are *vraSR*, *graSR,* and *walKR,* and the *rpoB* gene (6). This chromosomal mutation leads to the upregulation of peptidoglycan biosynthesis and cell wall thickening and further prevents vancomycin from reaching its target (7). Multiple nonsynonymous mutations were seen in the *vraSR*, *graSR,* and *walKR* TCSs of all sequenced isolates. In addition, a mutation was seen in a teicoplanin resistance-associated (*tcaRAB*) operon. A mutation in the *rpoB* gene was not observed in any of the sequenced isolates.

Taken together, comparative genomic analysis of these sequenced isolates revealed preferential clustering of single nucleotide polymorphisms (SNPs) in hVISA candidate genes with high diversity across the loci of *vraSR*, *graSR*, *walKR*, and *tcaRAB*.

### Accession number(s).

The draft genome sequences have been deposited in DDBJ/ENA/GenBank under the accession numbers as provided in Table 1.

## References

[1] Charles PG, Ward PB, Johnson PD, Howden BP, Grayson ML 2004 Clinical features associated with bacteremia due to heterogeneous vancomycin-intermediate *Staphylococcus aureus*. Clin Infect Dis 38:448–451. doi:10.1086/381093. 14727222

[2] Fridkin SK, Hageman J, McDougal LK, Mohammed J, Jarvis WR, Perl TM, Tenover FC, Vancomycin-Intermediate Staphylococcus aureus Epidemiology Study Group 2003 Epidemiological and microbiological characterization of infections caused by *Staphylococcus aureus* with reduced susceptibility to vancomycin, United States, 1997–2001. Clin Infect Dis 36:429–439. 12567300 10.1086/346207

[3] Wattam AR, Abraham D, Dalay O, Disz TL, Driscoll T, Gabbard JL, Gillespie JJ, Gough R, Hix D, Kenyon R, Machi D, Mao C, Nordberg EK, Olson R, Overbeek R, Pusch GD, Shukla M, Schulman J, Stevens RL, Sullivan DE, Vonstein V, Warren A, Will R, Wilson MJC, Yoo HS, Zhang C, Zhang Y, Sobral BW 2014 PATRIC, the bacterial bioinformatics database and analysis resource. Nucleic Acids Res 42:D581–D591. doi:10.1093/nar/gkt1099. 24225323 PMC3965095

[4] Tatusova T, DiCuccio M, Badretdin A, Chetvernin V, Ciufo S, Li W 2013 Prokaryotic genome annotation pipeline. The NCBI handbook, 2nd ed. National Center for Biotechnology Information, Bethesda, MD.

[5] Zankari E, Hasman H, Cosentino S, Vestergaard M, Rasmussen S, Lund O, Aarestrup FM, Larsen MV 2012 Identification of acquired antimicrobial resistance genes. J Antimicrob Chemother 67:2640–2644. doi:10.1093/jac/dks261. 22782487 PMC3468078

[6] Hafer C, Lin Y, Kornblum J, Lowy FD, Uhlemann AC 2012 Contribution of selected gene mutations to resistance in clinical isolates of vancomycinintermediate *Staphylococcus aureus*. Antimicrob Agents Chemother 56:5845–5851. doi:10.1128/AAC.01139-12. 22948864 PMC3486570

[7] Howden BP, Davies JK, Johnson PD, Stinear TP, Grayson ML 2010 Reduced vancomycin susceptibility in *Staphylococcus aureus*, including vancomycin-intermediate and heterogeneous vancomycin-intermediate strains: resistance mechanisms, laboratory detection, and clinical implications. Clin Microbiol Rev 23:99–139. doi:10.1128/CMR.00042-09. 20065327 PMC2806658

